# Affective evaluation of errors and neural error processing in obsessive-compulsive disorder

**DOI:** 10.1093/scan/nsad022

**Published:** 2023-04-25

**Authors:** Luisa Balzus, Franziska Jüres, Norbert Kathmann, Julia Klawohn

**Affiliations:** Department of Psychology, Humboldt-Universität zu Berlin, Berlin, Germany; Berlin School of Mind and Brain, Humboldt-Universität zu Berlin, Berlin 10099, Germany; Department of Psychology, Humboldt-Universität zu Berlin, Berlin, Germany; Department of Psychology, Humboldt-Universität zu Berlin, Berlin, Germany; Department of Psychology, Humboldt-Universität zu Berlin, Berlin, Germany; Department of Medicine, MSB Medical School Berlin, Berlin 14197, Germany

**Keywords:** obsessive-compulsive disorder, event-related potentials, error monitoring, affective processing, error-related negativity

## Abstract

Even though overactive error monitoring, indexed by enhanced amplitudes of the error-related negativity (ERN), is a potential biomarker for obsessive-compulsive disorder (OCD), the mechanisms underlying clinical variations in ERN amplitude remain unknown. To investigate whether ERN enhancement in OCD results from altered error evaluation, we examined the trial-wise valence evaluation of errors and its relation to the ERN in 28 patients with OCD and 28 healthy individuals. Electroencephalogram was recorded during an affective priming paradigm in which responses in a go/no-go task were followed by valence-based word categorization. Results indicated that errors were followed by faster categorization of negative than positive words, confirming that negative valence is assigned to errors. This affective priming effect was reduced in patients with OCD, while go/no-go performance was comparable between groups. Notably, this reduction amplified with increasing symptom severity. These results suggest attenuated affective error evaluation in OCD, possibly resulting from interfering effects of anxiety. There was no evidence for a trial-level association between valence evaluation and ERN, implying that ERN amplitude does not reflect valence assignment to errors. Consequently, altered error monitoring in OCD may involve alterations in possibly distinct processes, with weaker assignment of negative valence to errors being one of them.

In our complex environment, errors provide important information to adjust behavior. However, since consequences can be severe, errors are aversive, distressing events (e.g., Hajcak and Foti, 2008). Affect and motivation thus play a growing role in research on error processing, yet little is known about affective processes that accompany error processing and their relation to neural signals. In this preregistered study (https://osf.io/j28hr/), we investigated the affective evaluation of errors and its relation to neural correlates of error processing in healthy individuals and patients with obsessive-compulsive disorder (OCD), a psychiatric disorder associated with overactive error monitoring (Riesel, 2019). Thereby, we aimed to shed light on the mechanism underlying overactive error monitoring in OCD and enhance our understanding of pathomechanisms involved in disorder development and maintenance.

Core symptoms of OCD, i.e., recurrent intrusive thoughts and repetitive behaviors, have been linked to an overactive error monitoring system. In line with this, studies using event-related potentials (ERPs) repeatedly report enhanced amplitudes of the error-related negativity (ERN) during simple response-conflict tasks in OCD (for meta-analysis, see Riesel, 2019). The ERN is a fronto-central negative deflection that is maximal approximately 50 ms after erroneous responses (Gehring *et al.*, 1993). The exact functional significance of the ERN remains debated. Several theories have been proposed, many interpreting the ERN as a component signaling the need for behavioral adjustment and recruitment of cognitive control to prevent future errors (Botvinick *et al.*, 2001; Holroyd and Coles, 2002). This predominantly cognitive view, however, does not take into account that errors elicit feelings of distress (Spunt *et al.*, 2012) and physiological responses associated with defensive mobilization (Hajcak and Foti, 2008; Hajcak *et al.*, 2003). Furthermore, experimental manipulations heightening the motivational significance of errors, e.g., punishment of errors (Riesel *et al.*, 2012) or critical evaluation of performance (Hajcak *et al.*, 2005), increase the ERN. Moreover, enhanced ERN amplitudes have been linked to high levels of anxiety, worry, and internalizing psychopathology, such as OCD and anxiety disorders (for reviews, see Riesel, 2019; Weinberg *et al.*, 2015). Therefore, theories have emerged that interpret the ERN as an alarm signal reflecting the emotional significance of errors (Luu *et al.*, 2000; Proudfit *et al.*, 2013).

Although ample evidence indicates that affective processes accompany error monitoring, little research has examined how the ERN relates to affective processing. Aarts *et al.* (2013) provided first evidence for a direct link between the ERN and valence evaluation of errors. To capture valence evaluation, they used an affective priming paradigm in which each response in a go/no-go task was followed by an affective word that participants were asked to categorize as positive or negative. They observed that after erroneous responses to no-go stimuli, participants categorized negative words faster and more accurately than positive words. After fast correct responses to go stimuli, participants categorized positive words faster than negative words. These findings suggest that affective valence is automatically assigned to own actions, with errors being appraised as negative and correct actions as positive. Several studies reported such response facilitation to words that are affectively congruent to the preceding action, referred to as affective priming effect, an index of the valence evaluation of actions (Aarts *et al.*, 2012; Balzus *et al.*, 2021). Notably, Aarts *et al.* (2013) observed that across participants, a larger delta ERN (i.e., larger amplitude difference between errors and correct responses) was associated with a larger affective priming effect (i.e., greater valence discrimination between errors and correct responses). For ERN alone, a trend-level association with the priming effect was found. These findings corroborate the idea that the ERN reflects the emotional significance of errors.

Such insights into the relation between ERN and affective processing may shed light on the mechanisms underlying overactive error monitoring in internalizing psychopathology. Although ERN enhancement is considered a biomarker for OCD and appears to play a central role in its pathophysiology (Riesel, 2019), the mechanisms contributing to heightened ERN magnitude remain unknown. For instance, it is unclear whether ERN enhancement in OCD results from altered affective processing of errors. Possibly, increased ERN amplitudes in OCD reflect enhanced emotional significance of errors that results from heightened harm-avoidant motivation (Riesel *et al.*, 2019b). In contrast, a key characteristic of OCD, heightened trait anxiety, has been linked to diminished affective error evaluation. Using an affective priming paradigm (based on Aarts *et al.*, 2012), we therefore investigated the affective evaluation of errors and its relation to the ERN in patients with OCD and healthy individuals.

In line with previous findings (Aarts *et al.*, 2012, 2013; Balzus *et al.*, 2021), we expected that errors are evaluated as negative, such that after erroneous responses to no-go stimuli (false alarms), participants across groups would categorize negative words faster and more accurately than positive words. To investigate whether the ERN encodes the affective valence of errors, we examined the trial-by-trial relation between ERN and error evaluation. Based on prior findings (Aarts *et al.*, 2013), we assumed that strong negative evaluation of errors relates to increased ERN amplitudes. Accordingly, we hypothesized that larger ERN amplitudes would be associated with larger response facilitation to negative compared to positive words after false alarms (i.e., a larger priming effect). To evaluate this hypothesis, we examined word categorization after false alarms using a linear mixed model (LMM) to predict the word categorization response time (RT) in each trial as a function of word valence (contrast positive − negative) and the single-trial ERN amplitude. A significant negative interaction between word valence and ERN would indicate that larger ERN amplitudes are associated with faster categorization of negative words relative to positive words. Such brain–behavior relation on a trial-by-trial level would provide direct evidence that the ERN reflects the affective evaluation of errors and enhance our understanding of the functional significance of the ERN.

We additionally explored the affective evaluation of correct actions. Correct responses are associated with an ERP component that resembles the ERN but is smaller, the correct-response negativity (CRN; Ford, 1999). Evidence suggests that the CRN may be enhanced in OCD, but findings are inconsistent and the functional significance of this component is not well understood (for review, see Michael *et al.*, 2021). Therefore, we examined the affective evaluation of correct actions and its relation to the CRN. We predicted that after correct responses to go stimuli, participants across groups would categorize positive words faster than negative words, indicating that correct actions are evaluated as positive. Moreover, we expected that this response facilitation would be associated with the CRN (Aarts *et al.*, 2013), suggesting a relation between action evaluation and CRN.

With regard to group differences, we hypothesized that patients with OCD differ from healthy individuals in the affective evaluation of their errors. Previous findings lead to contradictory predictions in this regard. On the one hand, ERN enhancement is frequently reported in OCD (Riesel, 2019) and is assumed to reflect heightened emotional significance of errors (Aarts *et al.*, 2013). Thus, the assignment of negative valence to errors could be enhanced in OCD, as indexed by a larger affective priming effect. This finding would corroborate the assumption that ERN enhancement in OCD results from altered affective evaluation of errors. On the other hand, trait anxiety, and specifically its worry component, appears to be associated with an attenuated valence evaluation of actions (Aarts *et al.*, 2013; De Saedeleer and Pourtois, 2016). Given that anxiety and worry are key characteristics of OCD (Dar and Iqbal, 2015), the assignment of negative valence to errors could be diminished in OCD, as indexed by a smaller affective priming effect. This finding would suggest that altered error evaluation may not contribute to ERN enhancement in OCD. We tested which of these predictions can be substantiated and explored effects of trait anxiety, worry, and OCD symptom severity on action evaluation.

## Method

### Participants

The sample size was planned based on a power analysis (see Supplementary Material). The sample included 30 patients with OCD and 30 healthy control participants individually matched for age, gender, and educational level. Two patients were excluded along with their matched control participants due to meeting preregistered exclusion criteria of a comorbid bipolar disorder (*n* = 1) or fewer than 10 false alarms (*n *= 1). This resulted in a final sample of 28 patients with OCD (*M*_age_ = 33.29, 17 females) and 28 control participants (*M*_age_ = 33.07, 17 females; Table 1), described previously with respect to a different task (Balzus *et al.*, 2022). Although the target sample of 30 participants per group was not reached, the final sample still provided adequate power (> 80%, see Supplementary Material). Patients were diagnosed using the Structured Clinical Interview for Diagnostic and Statistical Manual of Mental Disorders, Fourth Edition (SCID-I; Wittchen *et al.*, 1997) and recruited from the outpatient clinic for OCD at Humboldt-Universität zu Berlin. Details on current treatment, medication, and comorbidities are provided in the Supplementary Material. Control participants were recruited via online advertisement.

**Table 1.** Demographic and clinical characteristics in the groups of patients with OCD and healthy control participants

Characteristic	Patients with OCD (*n* = 28)	Healthy control participants (*n* = 28)	Test statistic^a^	*p*
Age (years)^b^	33.29 (8.57)	33.07 (8.20)	*t*(53.90) = −0.10	.924
Gender (*n* female:male)	17:11	17:11	χ^2^(1) = 0.00	1.000
Handedness (*n* right:left:ambidextrous)^c^	26:1:1	27:1:0	–^d^	1.000
Years of education^e^	12.14 (1.46)	12.14 (1.08)	*t*(49.74) = 0.00	1.000
Verbal intelligence (WST)	104.93 (7.14)	105.71 (8.30)	*t*(52.81) = 0.38	.706
BDI-II	14.14 (11.34)	1.86 (2.69)	*t*(30.03) = −5.58	<.001
OCI-R	25.75 (9.95)	6.25 (5.65)	*t*(42.75) = −9.02	<.001
PSWQ	61.21 (10.82)	37.07 (9.86)	*t*(53.54) = −8.73	<.001
STAI trait	53.29 (10.08)	32.29 (7.22)	*t*(48.92) = −8.96	<.001
Y-BOCS total score	23.36 (3.84)			
Y-BOCS obsessions	11.43 (1.81)			
Y-BOCS compulsions	11.86 (2.53)			

*Note:* Means are reported with standard deviations in parentheses (except for gender and handedness). This table is adapted from Balzus *et al.* (2022). WST = Wortschatztest; BDI-II = Beck Depression Inventory-II; OCI-R = Obsessive-Compulsive Inventory-Revised; PSWQ = Penn State Worry Questionnaire; STAI = State-Trait Anxiety Inventory; Y-BOCS = Yale-Brown Obsessive Compulsive Scale.

a Welch’s *t* test was used for continuous variables.

b The age range was 18–55 years (control participants: 20–54 years; patients with OCD: 18–55 years).

c Handedness was assessed with the Edinburgh Handedness Inventory (Oldfield, 1971).

d For Fisher’s exact test, there is no test statistic to report.

e Years of education include primary and secondary education.

All participants were native German speakers and had normal or corrected-to-normal vision. Exclusion criteria were (i) lifetime diagnosis of bipolar, psychotic, or substance-related disorders; (ii) neurological diseases; and (iii) the use of neuroleptic medication in the last 3 months or benzodiazepines in the last week. Additional exclusion criteria for control participants were any current or past psychiatric disorder or psychotherapeutic treatment. The study was approved by the local ethics committee at Humboldt-Universität zu Berlin and conducted in accordance with the Declaration of Helsinki. Participants provided written informed consent and were compensated with money or course credit.

### Self-report questionnaires

Participants completed the German version of the State-Trait Anxiety Inventory (STAI; Laux *et al.*, 1981; Spielberger *et al.*, 1983), Obsessive-Compulsive Inventory-Revised (OCI-R; Foa *et al.*, 2002; Gönner *et al.*, 2008), Beck Depression Inventory-II (BDI-II; Beck *et al.*, 1996; Hautzinger *et al.*, 2006), and Penn State Worry Questionnaire (PSWQ; Glöckner-Rist and Rist, 2014; Meyer *et al.*, 1990). We further administered a standardized German vocabulary test (Wortschatztest [WST]; Schmidt and Metzler, 1992) that allows estimation of verbal intelligence. Patients’ OCD symptom severity was assessed using the Yale-Brown Obsessive Compulsive Scale (Y-BOCS; Goodman *et al.*, 1989; Hand and Büttner-Westphal, 1991). Patients reported higher levels of obsessive-compulsive (OCI-R) and depressive (BDI-II) symptom severity, trait anxiety (STAI), and worry (PSWQ) than control participants (Table 1).

### Task

**Fig. 1.**
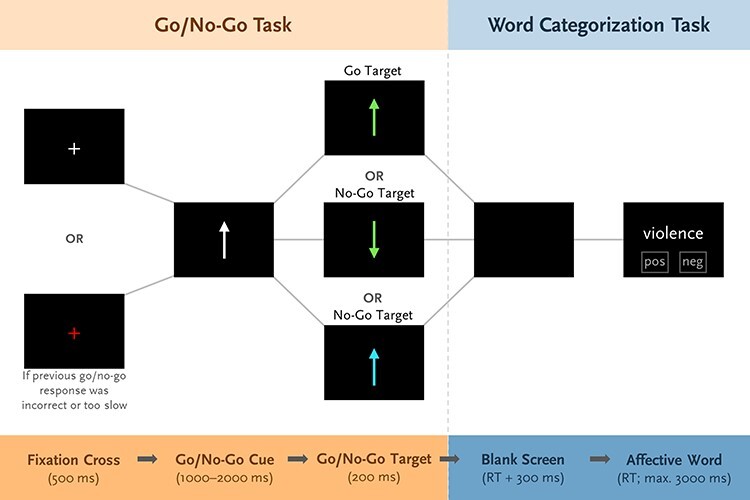
Trial sequence in the go/no-go task with the embedded word categorization task.

*Note:* A fixation cross was presented at the beginning of each trial, followed by a white upward- or downward-pointing arrow. Afterward, a green or turquoise arrow that pointed in the same or opposite direction as the white arrow was displayed. Participants were instructed to respond by key press if the white arrow turned green and kept the same direction and to withhold their response if it turned turquoise or changed its direction. Afterward, a blank screen was presented, followed by an affective word that participants were instructed to categorize as positive or negative. After the word categorization, the next trial started with a fixation cross. The cross was presented in red if the preceding go/no-go response was incorrect or above the individual RT limit and otherwise in white.

Participants performed a go/no-go task with an embedded word categorization task in which each go/no-go response was followed by the categorization of a word (Figure 1; see also Balzus *et al.*, 2021). This task was part of a project on the effects of transcranial direct current stimulation on error monitoring in OCD (Balzus *et al.*, 2022), comprising one session with active and one with placebo stimulation in randomized counterbalanced order. Only data obtained after placebo stimulation were included in the present study.

Each trial began with a fixation cross presented for 500 ms, followed by a white upward- or downward-pointing arrow for a variable interval of 1000 to 2000 ms. Afterward, a green arrow oriented in the same direction (go trial), a green arrow oriented in the opposite direction (no-go trial), or a turquoise arrow oriented in the same direction (no-go trial) was presented for 200 ms. Participants were instructed to press a key (go/no-go key) with their left index finger in go trials and to withhold response in no-go trials. Following the arrow, a blank screen was displayed and remained for 300 ms after key press or a maximum of 1800 ms. Afterward, an affective word was presented that participants categorized as positive or negative by pressing one of the two keys that were right to the go/no-go key with their right index or middle finger. Valence assignment to keys was counterbalanced across participants and shown as a reminder below each word. The word was displayed until response or for a maximum of 3000 ms.

Stimuli comprised 30 positive and 30 negative German words (Supplementary Table S1) from the Berlin Affective Word List Reloaded (Võ *et al.*, 2009). Positive and negative words did not differ in valence intensity, arousal, frequency, imageability, and the number of letters, syllables, and orthographic neighbors, |*t*|(58) < 1, *p* ≥ .353. Words were presented randomly, each appearing nine times without direct repetition.

The task was administered using Presentation software (Neurobehavioral Systems, Albany, CA, USA) and lasted approximately 35 min. Participants were instructed to respond as quickly and accurately as possible. They performed 24 practice trials. The task comprised 516 trials in three calibration blocks (28 trials: 20 go and 8 no-go) and six experimental blocks (72 trials: 48 go and 24 no-go) separated by breaks. Two experimental blocks followed each calibration block. Unknown to participants, calibration blocks were used for determining the individual RT limit in the go/no-go task to ensure sufficient false alarms. The limit was 80% (for the first two experimental blocks) or 90% (for all other experimental blocks) of the mean RT in the previous calibration block (limit range: 195–442 ms for control participants; 218–434 ms for patients with OCD). Accordingly, different response types were defined: fast hits (correct responses to go stimuli below RT limit), slow hits (correct responses to go stimuli above RT limit), inhibited responses (correct inhibitions to no-go stimuli), and false alarms (erroneous responses to no-go stimuli). Fast and slow hits were differentiated because particularly fast hits are evaluated positively and thus elicit a pronounced priming effect (Aarts *et al.*, 2012).

The fixation cross color at trial start provided feedback on the previous go/no-go response. It was red after incorrect responses and slow hits, and otherwise white. Additionally, written feedback was presented after each block, instructing participants to respond faster if false alarms occurred rarely (< 10%) and more accurately if they occurred frequently (> 50%). Otherwise, neutral feedback to continue responding quickly and accurately was presented. Before calibration blocks, feedback was neutral.

### EEG recording and preprocessing

Electroencephalogram (EEG) was recorded using a BrainAmp amplifier (BrainProducts, Gilching, Germany) from 25 Ag/AgCl electrodes placed according to the extended 10–20 system. Additional electrodes were placed at IO1 for recording the vertical electrooculogram, below T1 serving as the ground, and on both mastoids with the right mastoid serving as the recording reference. Impedances were kept below 5 kΩ. The signal was band-pass filtered at 0.01–250 Hz and sampled at 1000 Hz.

Data were processed offline using MATLAB (version 2019b) and the toolboxes EEGLAB (version 2019.1; Delorme and Makeig, 2004) and ERPLAB (version 8.01; Lopez-Calderon and Luck, 2014). Signals were filtered with a band-pass filter of 0.1–30 Hz (12 dB/octave roll-off) and a notch filter at 50 Hz, and re-referenced to the linked mastoids. After downsampling to 500 Hz, independent component analysis (Jung *et al.*, 2000) was used to correct eye movement artifacts. Epochs of 1500 ms locked to go/no-go responses with a 500 ms pre-response interval were extracted and baseline corrected using the 200 ms pre-response interval. We discarded epochs with a voltage difference exceeding 200 μV within an epoch or 50 μV between sample points, resulting in rejection of 0.58% (*SD* = 1.21) of epochs on average (remaining trials per participant for ERN analysis: *M* = 44.71, *SD* = 23.65; for CRN analysis: *M* = 333.21, *SD* = 17.67). To quantify single-trial ERN and CRN, we computed mean amplitudes from 0 to 100 ms post-response at FCz. Internal consistency was good for ERN (*r* = .89, 95% confidence interval [CI] = [.82, .93]) and excellent for CRN (*r* = .98, 95% CI [.98, .99]), as assessed with a permutation-based split-half method using 5000 random splits (splithalf package; version 0.8.2; Parsons, 2021) and Spearman–Brown correction.

### Statistical analysis

We performed LMMs on single-trial data using R (version 3.6.1). Trials were discarded (*M* = 8.72%, *SD* = 3.62) if the RT in the go/no-go task was < 100 ms or > 800 ms, the word categorization RT deviated more than three median absolute deviations from the individual condition-specific median, the response was missing, or the response was made with a key not assigned to the current task. In the analysis of word categorization RT, we additionally excluded incorrect categorizations.

Models were constructed using the lme4 package (version 1.1-25; Bates *et al.*, 2015b), and *p* values were obtained with the lmerTest package (version 3.1-3; Kuznetsova *et al.*, 2017) using Satterthwaite approximation. The significance level was *p* < .05. Analysis of RT, accuracy, and ERP amplitudes in the go/no-go task included group, and for RT also response type, as fixed effect. Word categorization RT and accuracy were modeled using the type of the preceding go/no-go response (slow hit, fast hit, false alarm, and inhibited response), word valence, and group as fixed effects. RT was log-transformed to normalize residuals. Accuracy was analyzed with a binomial generalized linear mixed model (GLMM). For testing brain–behavior relations, the single-trial within-participant *z*-standardized ERP was added as a predictor. To explore effects of OCD-related characteristics on action evaluation, we included group-mean-centered characteristics as predictors.

We used sliding difference contrasts for categorical fixed effects and started with the maximal random-effects structure for each model, with random intercepts for participants and (where applicable) words and random slopes for all fixed effects and their interactions. In case of non-convergence, random effects were specified as uncorrelated. Using principal component analysis, we identified random effects explaining zero variance and removed these, as recommended by Bates *et al.* (2015a). Significant and hypothesis-relevant interactions were followed up with *post hoc* comparisons using false discovery rate adjustment. Data and code are available at https://osf.io/j28hr.

## Results

Descriptive statistics for behavioral and ERP measures are provided in Supplementary Table S2. Statistical results of all analyses are reported in Tables 2–6. Results regarding the primary hypotheses are additionally presented in the main text. Effect sizes are reported as unstandardized regression coefficients (*b*) and odds ratios with 95% CIs.

**Table 2.** LMM results for RT and GLMM results for accuracy in the go/no-go task

	RT				Accuracy			
Fixed effect	*b*	95% CI	*t*	*p*	OR	95% CI	*z*	*p*
Intercept	5.73	[5.69, 5.76]	300.78	**<.001**	11.57	[9.88, 13.54]	30.45	**<.001**
FH − SH	−0.26	[−0.28, −0.24]	−24.82	**<.001**				
FA − FH	0.04	[0.02, 0.07]	3.41	**.001**				
Group (OCD − HC)	0.01	[−0.07, 0.09]	0.26	.797	1.03	[0.75, 1.41]	0.19	.848
FH − SH × Group	0.01	[−0.03, 0.05]	0.37	.710				
FA − FH × Group	−0.02	[−0.07, 0.03]	−0.93	.359				

*Note:* Estimates of the random effects are presented in Supplementary Table S3. Estimates of the model on RT (regression coefficients *b*) are on the log scale. Estimates of the model on accuracy reflect the probability of a correct response as odds ratios, and *p* values were calculated using Wald *Z* tests. Boldface *p* values represent statistical significance (*p* < .05). No. of observations: 21 164 (RT) and 27 892 (accuracy). FH = fast hit; SH = slow hit; FA = false alarm; HC = healthy control.

### Behavioral performance in the go/no-go task

RTs were shorter for fast hits than for slow hits and false alarms (Table 2). There were no significant group differences in RT and accuracy.

### Behavioral performance in the word categorization task

#### Response time

RT analysis yielded main effects of response type (Table 3). After false alarms, participants across groups categorized words slower than after fast hits, indicating post-error slowing. Moreover, word categorization after fast hits was faster than after slow hits.

**Table 3.** LMM results for word categorization RT and GLMM results for word categorization accuracy

	Word categorization RT				Word categorization accuracy			
Fixed effect	*b*	95% CI	*t*	*p*	OR	95% CI	*z*	*p*
Intercept	6.44	[6.39, 6.49]	272.84	**<.001**	21.38	[16.82, 27.18]	25.02	**<.001**
FH − SH	−0.02	[−0.03, −0.01]	−3.05	**.003**	0.87	[0.76, 1.00]	−1.92	.055
FA − FH	0.17	[0.13, 0.21]	8.42	**<.001**	0.68	[0.52, 0.88]	−2.89	**.004**
IR − FA	−0.02	[−0.06, 0.03]	−0.75	.457	2.92	[2.26, 3.78]	8.17	**<.001**
Valence (Pos − Neg)	0.01	[−0.02, 0.04]	0.83	.410	0.55	[0.37, 0.80]	−3.13	**.002**
Group (OCD − HC)	0.04	[−0.05, 0.13]	0.85	.402	1.30	[0.86, 1.96]	1.25	.213
FH − SH × Valence	−0.02	[−0.04, −0.00]	−2.13	**.038**	1.07	[0.81, 1.42]	0.46	.646
FA − FH × Valence	0.18	[0.13, 0.22]	8.15	**<.001**	0.10	[0.06, 0.18]	−7.75	**<.001**
IR − FA × Valence	−0.10	[−0.14, −0.06]	−5.29	**<.001**	5.44	[3.20, 9.26]	6.26	**<.001**
FH − SH × Group	0.01	[−0.01, 0.03]	1.11	.272	0.82	[0.63, 1.07]	−1.45	.147
FA − FH × Group	−0.02	[−0.09, 0.06]	−0.40	.688	0.92	[0.56, 1.52]	−0.34	.737
IR − FA × Group	−0.03	[−0.12, 0.06]	−0.57	.569	1.18	[0.71, 1.96]	0.65	.517
Valence × Group	−0.05	[−0.09, −0.00]	−2.17	**.034**	1.90	[1.05, 3.43]	2.13	**.033**
FH − SH × Valence × Group	0.00	[−0.03, 0.04]	0.05	.963	1.05	[0.62, 1.79]	0.20	.844
FA − FH × Valence × Group	−0.08	[−0.17, 0.01]	−1.87	.067	1.89	[0.62, 5.77]	1.12	.262
IR − FA × Valence × Group	0.06	[−0.02, 0.13]	1.52	.134	0.75	[0.26, 2.17]	−0.53	.599

*Note:* Estimates of the random effects are presented in Supplementary Table S4. Estimates of the model on RT (regression coefficients *b*) are on the log scale. Estimates of the model on accuracy reflect the probability of a correct response as odds ratios, and *p* values were calculated using Wald *Z* tests. Boldface *p* values represent statistical significance (*p* < .05). No. of observations: 24 189 (RT) and 26 123 (accuracy). FH = fast hit; SH = slow hit; FA = false alarm; IR = inhibited response; Pos = positive; Neg = negative; HC = healthy control.

The analysis further revealed significant two-way interactions between group and word valence and between each response type contrast and word valence (Table 3). To test our predictions regarding an affective priming effect, we followed up the latter interactions with *post hoc* comparisons (Table 4). These indicated that after false alarms, participants across groups categorized negative words faster than positive words (*b *= 0.12, 95% CI [0.08, 0.16], *t *= 6.29, *p *< .001). After fast and slow hits, participants categorized positive words faster than negative words (fast hits: *b *= −0.06, 95% CI [−0.09, −0.02], *t *= −3.30, *p *= .002; slow hits: *b *= −0.04, 95% CI [−0.07, 0.00], *t *= −2.16, *p *= .044).

**Table 4.** *Post hoc* comparisons based on models in Table 3 for the interactions between response type and word valence, and the interaction between the response type contrast false alarms − fast hits, word valence, and group

	Word categorization RT				Word categorization accuracy			
Fixed effect	*b*	95% CI	*t*	*p*	OR	95% CI	*z*	*p*
*Interactions FH − SH × Valence (Pos − Neg), FA − FH × Valence, and IR − FA × Valence*								
SH: Valence	−0.04	[−0.07, 0.00]	−2.16	**.044**	1.08	[0.68, 1.69]	0.32	.752
FH: Valence	−0.06	[−0.09, −0.02]	−3.30	**.002**	1.15	[0.72, 1.84]	0.58	.750
FA: Valence	0.12	[0.08, 0.16]	6.29	**<.001**	0.11	[0.07, 0.20]	−7.79	**<.001**
IR: Valence	0.02	[−0.02, 0.06]	0.99	.325	0.63	[0.36, 1.08]	−1.69	.182
*Interaction FA − FH × Valence × Group (OCD − HC)*								
FH: Valence × Group	−0.02	[−0.08, 0.04]	−0.67	.502	1.50	[0.68, 3.33]	1.01	.315
FH: HC: Valence	−0.05	[−0.09, 0.00]	−2.00	**.047**	0.94	[0.51, 1.73]	−0.21	.836
FH: OCD: Valence	−0.07	[−0.11, −0.02]	−2.92	**.008**	1.41	[0.76, 2.62]	1.08	.371
FA: Valence × Group	−0.10	[−0.17, −0.03]	−2.94	**.008**	2.84	[1.08, 7.46]	2.12	.067
FA: HC: Valence	0.17	[0.12, 0.22]	6.61	**<.001**	0.07	[0.03, 0.14]	−7.09	**<.001**
FA: OCD: Valence	0.07	[0.02, 0.12]	2.67	**.011**	0.19	[0.10, 0.39]	−4.52	**<.001**

*Note: Post hoc* comparisons were conducted using the emmeans package (version 1.6.0; Lenth, 2021). False discovery rate-adjusted *p* values are reported. Estimates of the model on RT (regression coefficients *b*) are on the log scale. Estimates of the model on accuracy reflect the probability of a correct response as odds ratios, and *p* values were calculated using Wald *Z* tests. Boldface *p* values represent statistical significance (*p* < .05). FH = fast hit; SH = slow hit; FA = false alarm; IR = inhibited response; Pos = positive; Neg = negative; HC = healthy control.

**Fig. 2.**
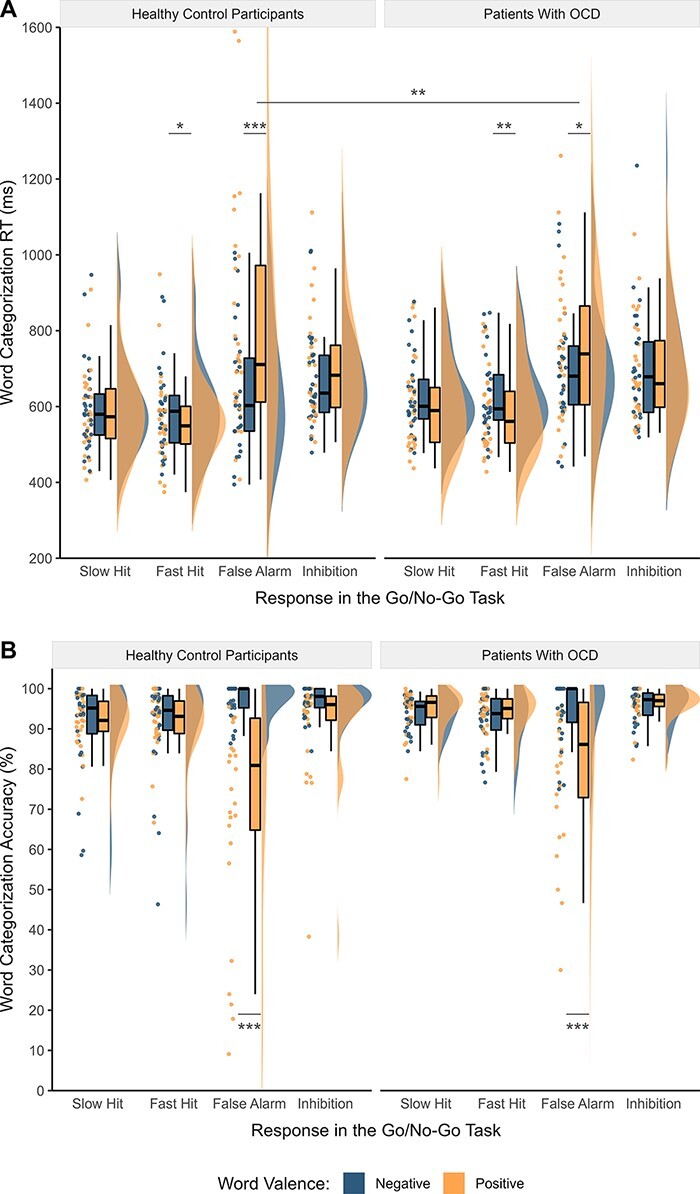
Observed word categorization RT and accuracy in patients with OCD and healthy control participants.

*Note:* (A) Observed word categorization RT per preceding response type in the go/no-go task, word valence, and group. (B) Observed word categorization accuracy per preceding response type in the go/no-go task, word valence, and group. (A–B) The plots show individual data points, boxplots, and probability density plots based on raw data that were aggregated by participant. Asterisks indicate significant differences yielded by the (G)LMM analyses. The plots were generated using the raincloudplots package (version 0.2.0; Allen *et al.*, 2021). *p < .05, **p < .01, ***p < .001.

**Fig. 3.**
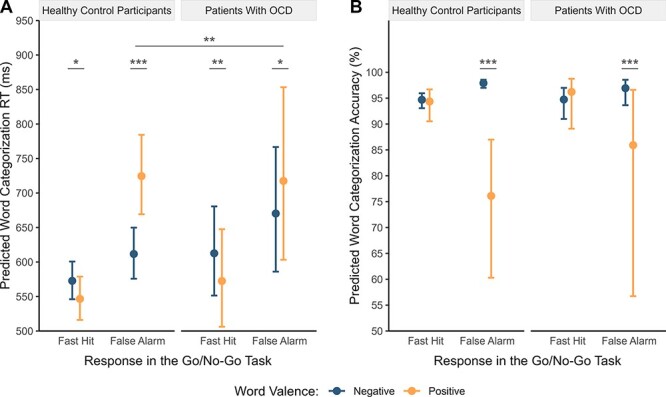
Model-predicted word categorization RT and accuracy in patients with OCD and healthy control participants.

*Note:* (A) Model-predicted mean word categorization RT per preceding response type (fast hit, false alarm) in the go/no-go task, word valence, and group, computed as partial effects from the LMM. RT data were log-transformed for analysis but back-transformed to ms for visualization. (B) Model-predicted mean word categorization accuracy per preceding response type (fast hit, false alarm) in the go/no-go task, word valence, and group, computed as partial effects from the GLMM. (A–B) Error bars indicate 95% CIs. Asterisks indicate significant differences yielded by the (G)LMM analyses. *p < .05, **p < .01, ***p < .001.

Crucially, the significant two-way interactions were qualified by a statistical trend for a three-way interaction between the response type contrast false alarms − fast hits, word valence, and group (Table 3). *Post hoc* comparisons indicated that after false alarms, both groups categorized negative words faster than positive words, whereas after fast hits, they categorized positive words faster than negative words (Table 4; Figures 2A and 3A). Comparisons conducted to investigate hypothesized group differences revealed that this response facilitation to negative relative to positive words after false alarms was significantly smaller in patients than in the control group (*b *= −0.10, 95% CI [−0.17, −0.03], *t *= −2.94, *p *= .008), indicating a reduced priming effect in OCD (Table 4).

#### Accuracy

Analysis of categorization accuracy revealed main effects of response type and word valence, which were qualified by significant interactions between response type and word valence and between group and word valence (Table 3). *Post hoc* comparisons indicated that after false alarms, participants across and within groups categorized negative words more accurately than positive words (across groups: odds ratio = 0.11, 95% CI [0.07, 0.20], *t *= −7.79, *p *< .001; Table 4; Figures 2B and 3B). This response facilitation to negative relative to positive words after false alarms was smaller in patients with OCD compared to control participants at the trend level (odds ratio = 2.84, 95% CI [1.08, 7.46], *t *= 2.12, *p *= .067).

### Electrophysiological results

**Fig. 4.**
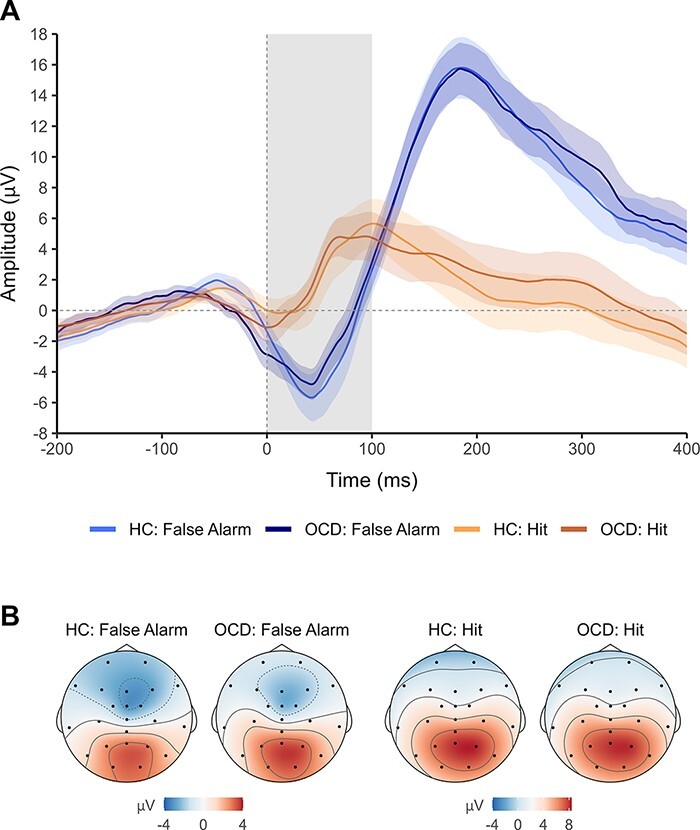
ERN and CRN in patients with OCD and healthy control (HC) participants.

*Note:* (A) Grand average response-locked waveforms at electrode site FCz with 95% CIs in patients with OCD and HC participants for correct (hit) and incorrect (false alarm) responses in the go/no-go task. The gray-shaded time interval was used to quantify ERN and CRN amplitudes. (B) Scalp topographies of ERN and CRN in both groups in the time window 0–100 ms after false alarms and hits, respectively. (A–B) The plots were generated using the eegUtils package (version 0.6.1; Craddock, 2021).

Figure 4 shows ERN and CRN waveforms. Contrary to our expectation, ERN and CRN amplitudes did not differ significantly between groups (Table 5).

**Table 5.** LMM results for the ERN and CRN in the go/no-go task

	ERN				CRN			
Fixed effect	*b*	95% CI	*t*	*p*	*b*	95% CI	*t*	*p*
Intercept	−2.65	[−3.93, −1.37]	−4.15	**<.001**	2.33	[1.41, 3.25]	5.07	**<.001**
Group (OCD − HC)	0.52	[−2.04, 3.09]	0.41	.684	0.05	[−1.79, 1.89]	0.05	.956

*Note:* Models on ERN and CRN included false alarm trials and hit trials, respectively. Estimates of the random effects are presented in Supplementary Table S8. Estimates of the fixed effects (regression coefficients *b*) directly reflect mean differences in μV. Boldface *p* values represent statistical significance (*p* < .05). No. of observations: 2504 (ERN) and 18 660 (CRN). HC = healthy control.

#### Relation between error evaluation and ERN amplitude

Analysis of word categorization RT in false alarm trials with ERN as a predictor yielded a main effect of word valence, with negative words being categorized faster than positive words, again revealing the priming effect after false alarms (Table 6). Contrary to our prediction, we found no significant interaction between ERN and word valence (*p* = .807). Hence, there was no evidence for a relation between error evaluation (indexed by the RT priming effect) and ERN. Furthermore, there was no difference in this relation between groups. There was also no correlation between the priming effect after false alarms and ERN across participants, *r*(54) = −.09, 95% CI [−.34, .18], *p *= .525.

**Table 6.** LMM results for word categorization RT with ERN and CRN as predictors

	Word categorization RT							
	With ERN as a predictor				With CRN as a predictor			
Fixed effect	*b*	95% CI	*t*	*p*	*b*	95% CI	*t*	*p*
Intercept	6.52	[6.46, 6.59]	197.70	**<.001**	6.36	[6.31, 6.40]	269.74	**<.001**
Valence (Pos − Neg)	0.12	[0.07, 0.18]	4.50	**<.001**	−0.06	[−0.08, −0.03]	−4.21	**<.001**
Group (OCD − HC)	0.05	[−0.09, 0.18]	0.68	.497	0.06	[−0.04, 0.15]	1.25	.216
ERP^a^	0.00	[−0.01, 0.01]	0.46	.646	−0.01	[−0.01, −0.00]	−2.04	**.046**
Valence × Group	−0.09	[−0.20, 0.01]	−1.75	.086	−0.03	[−0.07, 0.02]	−1.14	.257
Valence × ERP^a^	0.00	[−0.02, 0.02]	0.25	.807	−0.00	[−0.02, 0.01]	−0.84	.406
Group × ERP^a^	0.01	[−0.01, 0.03]	1.17	.243	−0.01	[−0.02, 0.00]	−1.58	.115
Valence × Group × ERP^a^	0.01	[−0.03, 0.05]	0.57	.571	0.01	[−0.01, 0.03]	1.10	.269

*Note:* ERN and CRN amplitudes were entered as *z*-standardized continuous predictors in the models including only false alarm trials and fast hit trials, respectively. Estimates of the random effects are presented in Supplementary Table S9. Estimates of the fixed effects (regression coefficients *b*) are on the log scale. Boldface *p* values represent statistical significance (*p* < .05). No. of observations: 1992 (model with ERN) and 5025 (model with CRN). Pos = positive; Neg = negative; HC = healthy control.

a ERP refers to ERN or CRN as a predictor in the respective model.

#### Relation between evaluation of correct actions and CRN amplitude

Analysis of word categorization RT in fast hit trials including CRN as a predictor indicated a main effect of word valence. This again demonstrated the priming effect after fast hits, with positive words being categorized faster than negative words (Table 6). Moreover, a main effect of CRN was found, with smaller CRN amplitude being related to faster RT. There were no significant interactions between CRN, word valence, and group. Thus, we found no evidence for a relation between action evaluation and CRN (*p* = .406) and no group difference in this relation. Also no correlation was observed between the priming effect after fast hits and CRN across participants, *r*(54) = .15, 95% CI [−.12, .40], *p *= .273.

### Effects of OCD-related characteristics on action evaluation

**Fig. 5.**
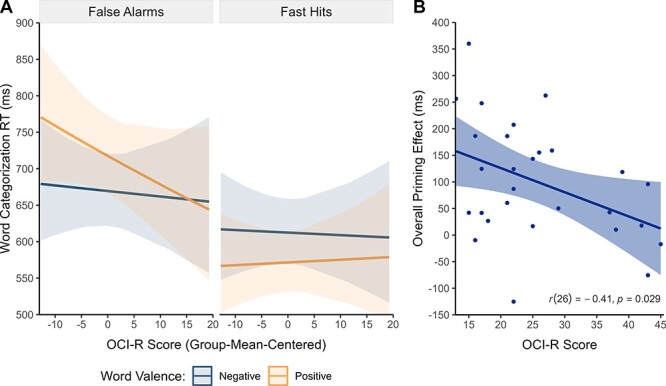
Association between symptom severity and the affective evaluation of actions in patients with OCD.

*Note:* (A) Model-predicted interaction effect between OCI-R score, preceding response type (fast hit, false alarm) in the go/no-go task, and word valence on word categorization RT in patients with OCD, computed as partial effects from the LMM. RT data were log-transformed for analysis but back-transformed to ms for data visualization. The plot was generated using the sjPlot package (version 2.8.11; Lüdecke, 2022). (B) Negative correlation between the OCI-R score and the overall priming effect across participants. The overall priming effect refers to the priming effect after false alarms and fast hits and was calculated by subtracting the RT in congruent conditions (positive words after fast hits and negative words after false alarms) from the RT in incongruent conditions (positive words after false alarms and negative words after fast hits) for each participant (Aarts *et al.*, 2012). (A–B) Shaded bands represent 95% CIs.

Exploratory analyses on the effects of trait anxiety, worry, and OCD symptom severity on action evaluation within both groups (Supplementary Tables S5–S7) revealed an interaction between OCD symptom severity, word valence, and the response type contrast false alarms − fast hits in the patient group (*b *= −0.01, 95% CI [−0.01, −0.00], *t *= −2.44, *p *= .021). Thus, higher symptom severity in patients was associated with a smaller overall priming effect after fast hits and false alarms (Figure 5). This finding was supported by a *post hoc* Pearson correlation analysis, which revealed a negative correlation between the overall priming effect and symptom severity in patients, *r*(26) = −.41, 95% CI [−.68, −.05], *p* = .029. A trend for a similar relation was observed for trait anxiety (*b *= −0.00, 95% CI [−0.01, 0.00], *t *= −2.03, *p *= .053), *r*(26) = −.34, 95% CI [−.63, .04], *p* = .078 (Supplementary Figure S1). There was no significant effect of trait worry.

## Discussion

In this study, we used an affective priming paradigm to investigate the valence evaluation of errors and its trial-by-trial relation to the ERN in patients with OCD and healthy individuals. Thereby, we aimed to examine whether ERN enhancement in OCD is linked to altered error evaluation. In support of our hypotheses, we found an affective priming effect after errors, an index of error evaluation, to be present in both groups yet significantly reduced in patients with OCD. Contrary to our hypothesis, no trial-by-trial association between error evaluation and ERN was evident.

The findings confirm predicted behavioral effects. After false alarms to no-go stimuli, participants across and within groups categorized negative words faster and more accurately than positive words. After fast hits to go stimuli, participants across and within groups categorized positive words faster than negative words. This corroborates previous evidence suggesting that affective valence is automatically assigned to own actions, with errors being appraised as negative and correct actions as positive (Aarts *et al.*, 2012, 2013; Balzus *et al.*, 2021). Our findings align with evidence indicating that errors elicit negative affect (for review, see Dignath *et al.*, 2020) and physiological responses that reflect defensive mobilization (Hajcak and Foti, 2008).

We observed a similar pattern of action evaluation in patients with OCD as in healthy individuals. However, as predicted, patients differed from healthy individuals in the magnitude of the priming effect after errors, while RT and error rate in the go/no-go task were comparable between groups. In line with one of the two contrary predictions based on previous findings, patients showed reduced priming after errors, evident as smaller RT facilitation to negative relative to positive words compared to healthy individuals. The same pattern was observed for accuracy at the trend level. These results indicate that patients with OCD show altered error evaluation. In particular, the assignment of negative valence to errors seems attenuated in OCD. In our study, the groups differed only in this specific aspect of error monitoring, one aspect that has not previously been investigated in OCD. Crucially, both exploratory LMM and correlation analyses indicated that within the patient group, higher levels of OCD symptoms and trait anxiety (at the trend level) were associated with attenuated action evaluation. These findings support the notion that OCD symptoms are linked to impairments in assigning valence to own actions.

The results align with evidence indicating that individuals with elevated levels of trait anxiety and worry show decreased valence evaluation of own actions (Aarts *et al.*, 2012; De Saedeleer and Pourtois, 2016). De Saedeleer and Pourtois (2016) presumed that worry interferes with action monitoring by modulating activity in parts of the rostral cingulate cortex, such as the anterior cingulate cortex (ACC; Shackman *et al.*, 2011), and/or limbic structures connected to it, such as the amygdala (Grupe and Nitschke, 2013). Accordingly, valence assignment to actions might be hampered in OCD because anxious and worrisome thoughts that are characteristic of these patients (as also evident in this study) may interfere with valence evaluation during action monitoring. This is consistent with the notion that trait anxiety and worry have detrimental effects on action monitoring (Eysenck *et al.*, 2007).

Contrary to our predictions, we observed no trial-by-trial association between error evaluation and ERN. Additionally, there was no evidence for a relation between the evaluation of correct actions and CRN. Aarts *et al.* (2013) reported trend-level associations between ERN (and CRN) and action evaluation across participants. These trend-level associations at the between-participants level could not be replicated in the present study with a larger sample. Moreover, our results did not confirm that the ERN encodes the valence evaluation of errors at the trial-by-trial level. A possible explanation can be derived from accounts interpreting the ERN as an alarm signal that reflects mobilization of defensive responses (Proudfit *et al.*, 2013). This interpretation of the ERN in the context of the absence of evidence for a relation between ERN and valence evaluation of errors potentially suggests that ERN and priming effect may be manifestations of distinct processes during error monitoring. Possibly playing different roles in guiding adaptive behavior, the ERN may indicate an initial assessment of the motivational salience of errors that is associated with defensive mobilization (Weinberg *et al.*, 2012), while the valence evaluation may reflect a subsequent evaluative judgment. Supporting this view, we previously found no relation between valence evaluation and skin conductance response, suggesting that valence evaluation and defensive mobilization operate independently during action monitoring (Balzus *et al.*, 2021).

Hence, our findings did not corroborate the assumption that often-replicated ERN enhancement in OCD reflects strong negative evaluation of errors. Rather, increased ERN amplitudes in OCD may result from enhanced motivational salience of errors (Endrass *et al.*, 2010; Riesel *et al.*, 2019a), possibly associated with defensive mobilization (Weinberg *et al.*, 2012) and caused by elevated harm-avoidant motivation (Riesel *et al.*, 2019b). Consequently, our findings can be interpreted within the framework by Proudfit *et al.* (2013). According to this framework, the ERN emerges as an immediate defensive response to errors, and ERN enhancement results from elevated threat sensitivity to errors. Heightened defensive mobilization, caused by enhanced threat sensitivity, is proposed to be associated with subsequent cognitive processes that may include detrimental processes, such as worry. In the context of this theory, these processes may divert cognitive resources from the valence evaluation of errors. Accordingly, in the present study, alterations in error evaluation were evident in OCD and were associated with symptom severity and trait anxiety, corroborating the idea that these alterations result from detrimental effects of anxiety and worry. Notably, we observed no evidence for an increased ERN in the patient group. This might be attributed to the complexity of the applied task, given that ERN group differences are mostly evident in simple response-conflict tasks (Riesel, 2019), or to the limited statistical power due to the relatively small sample.

Since our findings did not confirm that the ERN reflects the valence evaluation of errors, it is conceivable that heightened ERN amplitudes and diminished valence evaluation in OCD reflect co-occurring alterations in independently operating processes during error monitoring. Accordingly, patients with OCD seem to show weaker valence evaluation of errors, while they may exhibit normal (as in this study) or stronger (as evidenced by simple response-conflict tasks) defensive response that is presumably reflected in ERN amplitude. It is possible, however, that both error processing alterations result from the same underlying dysfunction of the ACC. The ACC, an integration hub for information from affective and cognitive structures (Shackman *et al.*, 2011), is considered the major source of ERN (Debener *et al.*, 2005) and action appraisal (Dixon *et al.*, 2017) and is overactive during error processing in OCD (Fitzgerald *et al.*, 2005; Ursu *et al.*, 2003). Hence, ACC hyperactivity may underlie multiple error processing alterations that manifest as enhanced ERN and reduced valence evaluation. Future research is needed to substantiate this hypothesis, possibly using functional neuroimaging and a simplified task in which both ERN enhancement and diminished error evaluation are evident.

Given the high task complexity in this study, other simultaneous task-related processes may have interfered with error monitoring, resulting in similar ERN amplitudes in both groups. Thus, our results are limited to conditions of high task complexity, and it cannot be excluded that different results are obtained with less complex tasks. Another limitation of the study is that it was part of a project comprising one session with active and one with placebo transcranial direct current stimulation in counterbalanced order, which preceded task performance. During both sessions, participants and experimenters were blind to the stimulation condition. In the present study, data from the placebo session were analyzed. Thus, measurements of participants who completed the task for the first or second time were included. Importantly, control analyses indicated that task familiarity (i.e., session number) did not impact hypothesis-relevant results (Supplementary Tables S10 and S11). Nevertheless, it cannot be excluded that task repetition affected behavioral and ERP measures.

In summary, our findings indicate that both healthy individuals and patients with OCD automatically assign affective valence to own actions. In OCD, valence assignment to errors seems attenuated, notably an effect that increases with symptom severity and possibly results from interfering effects of anxiety. We found no evidence that the ERN reflects the valence evaluation of errors, suggesting that ERN enhancement in OCD may not result from altered error evaluation. Accordingly, patients with OCD may show co-occurring alterations in potentially distinct subprocesses of error processing: weaker assignment of negative valence to errors (indicated by attenuated affective priming) and possibly stronger defensive response (typically reflected in ERN enhancement). Consequently, heightened harm-avoidant motivation in OCD may result in enhanced emotional significance of errors at the level of motivational salience rather than at the level of affective valence.

## Supplementary Material

nsad022_Supp

## Data Availability

The data underlying this article are available on the Open Science Framework at https://osf.io/j28hr.
