# Supplementary material for: Affective evaluation of errors and neural error processing in obsessive-compulsive disorder
Source: Soc Cogn Affect Neurosci. 2023 Apr 25;18(1):nsad022. doi: 10.1093/scan/nsad022 (PMC10243905; doi:10.1093/scan/nsad022)
Supplement: nsad022_Supp [file nsad022_supp.zip › scan-22-244-File002.docx]

**Affective Evaluation of Errors and Neural Error Processing**

**in Obsessive-Compulsive Disorder**

Luisa Balzus^1, 2*^, Franziska Jüres^1*^, Norbert Kathmann^1^, and Julia Klawohn^1, 3^

^1^ Humboldt-Universität zu Berlin, Department of Psychology, Berlin, Germany

^2^ Humboldt-Universität zu Berlin, Berlin School of Mind and Brain, Berlin, Germany

^3^ MSB Medical School Berlin, Department of Medicine, Berlin, Germany

*Luisa Balzus and Franziska Jüres contributed equally to this work and share first authorship.

Correspondence: [luisa.balzus@alumni.hu-berlin.de](mailto:luisa.balzus@alumni.hu-berlin.de)

**Supplementary Material**

In this Supplementary Material, additional information on the following topics is provided:

- a priori sample size determination (page 1),
- clinical characteristics in the group of patients with obsessive-compulsive disorder (OCD; page 2),
- stimulus material in the word categorization task (Table S1),
- descriptive statistics for behavioral performance and event-related potentials (Table S2),
- random effects of the reported linear mixed models (LMMs; Tables S3, S4, S8, and S9),
- results of exploratory LMM and correlation analyses on effects of OCD-related characteristics on action evaluation (Figure S1; Tables S5, S6, and S7), and
- results of control analyses on effects of task familiarity (i.e., session number) on action evaluation (Tables S10 and S11).

**Sample Size Determination**

As this study focuses on the affective evaluation of errors, we estimated the power to detect the predicted affective priming effect after false alarms (i.e., faster categorization of negative compared to positive words) and its interaction with the error-related negativity (ERN) separately in both groups. We conducted a simulation-based power analysis using the SIMR package (Version 1.0.5; Green & MacLeod, 2016) for LMMs in R (the code is available at https://osf.io/j28hr/). In the simulation, estimates of fixed effects not related to the hypotheses and estimates of random effects were based on data from a previous study in which the same task was administered to a sample of 30 healthy participants (Balzus et al., 2021). Since distribution of response time (RT) data is typically positively skewed, inverse transformed RT data and effect estimates were used in the simulations. The power analysis with 1,000 simulations indicated that with a sample of 30 participants, an RT facilitation of 40 ms (unstandardized coefficient of 0.09) for negative compared to positive words after false alarms would be detected with a power of 85.70% (95% confidence interval [CI] = [83.38, 87.81]) in both groups. This magnitude of RT facilitation is considerably smaller than previously reported (Aarts et al., 2012, 2013; Balzus et al., 2021). A relation between this RT facilitation and the *z*-standardized ERN of a coefficient of −0.05 would be detected with a power of 86.70% (95% CI [84.44, 88.74]). On the basis of this power analysis and also informed by the power analysis for a different set of research questions that were part of the overall project (Balzus et al., 2022), 30 patients with OCD and 30 healthy participants were enrolled in the study. Given that two patients were excluded with their matched control participants due to meeting preregistered exclusion criteria, the target sample of 30 participants per group was not reached. However, conductance of the power analysis described above with 28 participants per group indicated that the final sample still provided adequate power (for RT facilitation for negative compared to positive words after false alarms: 82.40%, 95% CI [79.90, 84.71]; for a relation between this RT facilitation and the *z*-standardized ERN: 82.20%, 95% CI [79.69, 84.52]).

**Clinical Characteristics in the Group of Patients With OCD**

Most patients were on a waiting list for cognitive behavioral therapy (CBT; *n* = 23), and some were currently receiving CBT (*n* = 5). Thirteen patients received medical treatment (selective serotonin reuptake inhibitors [SSRIs], *n* = 10; serotonin–norepinephrine reuptake inhibitors, *n* = 1; combination of SSRIs and other antidepressants, *n* = 2). Twenty patients were diagnosed with one or more of the following comorbid psychiatric disorders: major depressive disorder (current episode, *n* = 3; remitted, *n* = 10), dysthymia (*n* = 3), social phobia (*n* = 4), specific phobia (*n*= 2), generalized anxiety disorder (*n* = 1), panic disorder (*n* = 1), and bulimia nervosa (*n* = 1).

**Table S1**

*Word Stimuli in the Word Categorization Task*

| Positive words | | Negative words | |
| --- | --- | --- | --- |
| Feier *(celebration)* | Sieg *(victory)* | Armut *(poverty)* | Tyrann *(tyrant)* |
| Freiheit *(freedom)* | Spaß *(fun)* | Betrug *(fraud)* | Übelkeit *(nausea)* |
| Freude *(joy)* | Talent *(talent)* | Defizit *(deficit)* | Verbrechen *(crime)* |
| Geschenk *(gift)* | Triumph *(triumph)* | Gefängnis *(prison)* | Verlust *(loss)* |
| Gewinn *(gain)* | Vertrauen *(trust)* | Gestank *(stench)* | Versager *(loser)* |
| Glück *(luck)* | beliebt *(popular)* | Gewalt *(violence)* | bankrott *(bankrupt)* |
| Held *(hero)* | brillant *(brilliant)* | Gift *(poison)* | einsam *(lonely)* |
| Humor *(humor)* | genial *(ingenious)* | Grab *(grave)* | herzlos *(heartless)* |
| Jubel *(cheer)* | grandios *(magnificent)* | Krieg *(war)* | krank *(ill)* |
| Liebe *(love)* | kreativ *(creative)* | Opfer *(victim)* | lieblos *(loveless)* |
| Mut *(courage)* | optimal *(optimal)* | Pech *(misfortune)* | mies *(lousy)* |
| Party *(party)* | perfekt *(perfect)* | Plage *(nuisance)* | traurig *(sad)* |
| Reise *(journey)* | super *(super)* | Qual *(agony)* | trostlos *(dismal)* |
| Retter *(rescuer)* | toll *(great)* | Strafe *(punishment)* | unfair *(unfair)* |
| Schatz *(treasure)* | topfit *(in top form)* | Tod *(death)* | verlogen *(dishonest)* |

*Note.* English translations in parentheses.

**Table S2**

*Descriptive Statistics for Behavioral Task Performance and Event-Related Potential (ERP) Measures in the Groups of Patients With Obsessive-Compulsive Disorder (OCD) and Healthy Control Participants*

| Measure | | Patients with OCD *M* [95% CI] | Healthy control participants *M* [95% CI] |
| --- | --- | --- | --- |
| *Behavioral performance in the go/no-go task* | | | |
| Response time (ms) |  | |  |
| SH | | 363 [360, 365] | 362 [360, 365] |
| FH | | 297 [295, 298] | 283 [281, 285] |
| FA | | 285 [279, 290] | 296 [290, 302] |
| Proportion of responses (%) | |  |  |
| SH | | 44.77 [41.70, 47.85] | 46.37 [42.86, 49.88] |
| FH | | 21.23 [18.23, 24.24] | 19.69 [16.47, 22.91] |
| FA | | 9.14 [6.97, 11.30] | 8.76 [7.33, 10.19] |
| IR | | 23.34 [21.19, 25.49] | 23.74 [22.33, 25.15] |
| Missing response | | 1.03 [0.52, 1.54] | 1.17 [0.45, 1.89] |
| Wrong key response | | 0.49 [0.26, 0.71] | 0.27 [0.16, 0.38] |
| *Behavioral performance in the word categorization task* | | | |
| Response time (ms) | |  |  |
| Pos word after SH | | 595 [590, 600] | 573 [569, 578] |
| Neg word after SH | | 621 [616, 625] | 590 [585, 594] |
| Pos word after FH | | 596 [589, 602] | 584 [577, 592] |
| Neg word after FH | | 635 [628, 642] | 616 [608, 624] |
| Pos word after FA | | 737 [713, 760] | 763 [734, 792] |
| Neg word after FA | | 675 [658, 692] | 628 [613, 643] |
| Pos word after IR | | 696 [688, 704] | 702 [693, 711] |
| Neg word after IR | | 689 [681, 698] | 665 [657, 672] |
| Accuracy (%) | |  |  |
| Pos word after SH | | 94.86 [94.02, 95.70] | 91.66 [90.63, 92.69] |
| Neg word after SH | | 94.03 [93.12, 94.93] | 90.64 [89.60, 91.68] |
| Pos word after FH | | 94.18 [92.89, 95.47] | 91.95 [90.40, 93.49] |
| Neg word after FH | | 92.35 [90.86, 93.83] | 91.23 [89.69, 92.77] |
| Pos word after FA | | 79.21 [75.88, 82.55] | 69.01 [65.23, 72.80] |
| Neg word after FA | | 94.57 [92.59, 96.54] | 96.71 [94.99, 98.44] |
| Pos word after IR | | 96.43 [95.45, 97.42] | 92.06 [90.68, 93.44] |
| Neg word after IR | | 96.14 [95.10, 97.17] | 96.52 [95.53, 97.51] |
| *ERP measures* | | | |
| ERN (µV) | | −2.21 [−2.89, −1.53] | −2.15 [−2.90, −1.41] |
| CRN (µV) | | 2.38 [2.15, 2.61] | 2.33 [2.10, 2.55] |

*Note.* Means and confidence intervals (CIs) were obtained by averaging single-trial data. CIs are adjusted for within-participant comparisons using the method described by Morey (2008). Error-related negativity (ERN) and correct-response negativity (CRN) were measured as mean amplitude from 0 to 100 ms at electrode FCz after false alarms and hits, respectively. SH = slow hit; FH = fast hit; FA = false alarm; IR = inhibited response; Pos = positive; Neg = negative.

**Table S3**

*Random Effects (SDs) of the Linear Mixed Model on Response Time (RT) and the Generalized Linear Mixed Model on Accuracy in the Go/No-Go Task*

| Random effect | RT | Accuracy |
| --- | --- | --- |
| Participants (*N* = 56) |  |  |
| Intercept | 0.14 | 0.58 |
| FH − SH | 0.07 |  |
| FA − FH | 0.08 |  |
| Residual ^a^ | 0.18 |  |

*Note.* Models with the maximal random-effects structure were used. FH = fast hit; SH = slow hit; FA = false alarm.

^a^ A direct estimate of residual variance is not provided by binomial generalized linear mixed models.

**Table S4**
*Random Effects (SDs) of the Linear Mixed Model on Word Categorization Response Time (RT) and the Generalized Linear Mixed Model on Word Categorization Accuracy*

| Random effect | Word categorization RT | Word categorization accuracy |
| --- | --- | --- |
| Participants (*N* = 56) |  |  |
| Intercept | 0.17 | 0.72 |
| FH − SH | 0.03 | – |
| FA − FH | 0.14 | 0.53 |
| IR − FA | 0.16 | 0.44 |
| Valence (Pos − Neg) | 0.08 | 0.94 |
| FH − SH × Valence | 0.05 | – |
| FA − FH × Valence | 0.14 | 1.39 |
| IR − FA × Valence | 0.12 | 1.08 |
| Word stimuli (*N* = 60) |  |  |
| Intercept | 0.03 | 0.50 |
| FH − SH | 0.01 | 0.19 |
| FA − FH | 0.01 | 0.33 |
| IR − FA | – | 0.09 |
| Group (OCD − HC) | 0.01 | 0.33 |
| FH − SH × Group | – | – |
| FA − FH × Group | – | 0.17 |
| IR − FA × Group | 0.01 | 0.15 |
| Residual ^a^ | 0.18 |  |

*Note.* Random effects were specified as uncorrelated due to non-convergence of the models with the maximal random-effects structure. Random effects explaining zero variance were removed and are indicated by dashes. FH = fast hit; SH = slow hit; FA = false alarm; IR = inhibited response; Pos = positive; Neg = negative; OCD = obsessive-compulsive disorder; HC = healthy control.

^a^ A direct estimate of residual variance is not provided by binomial generalized linear mixed models.

**Figure S1**

*Association Between Trait Anxiety and the Affective Evaluation of Actions in Patients With Obsessive-Compulsive Disorder (OCD)*


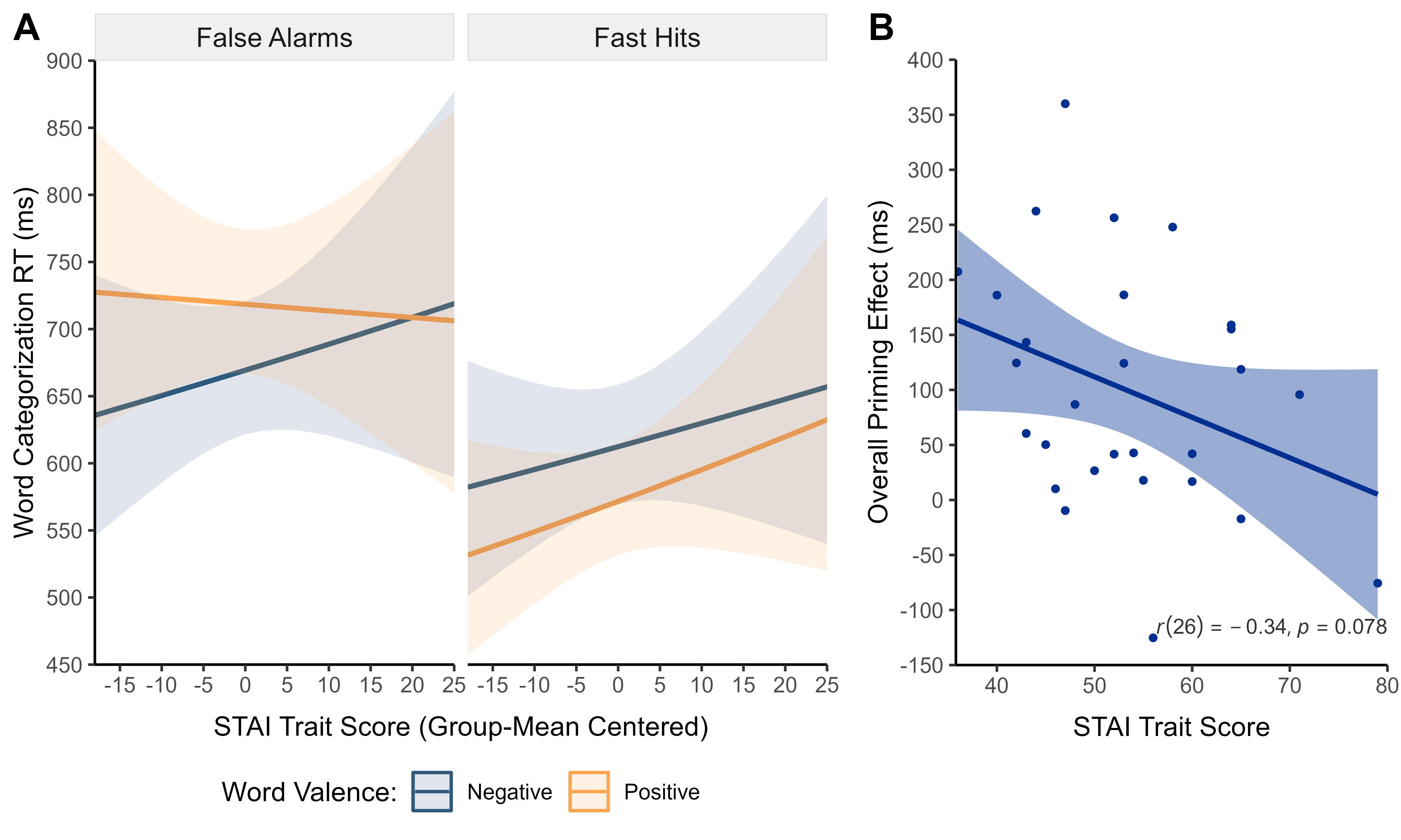


*Note.* **(A)** Model-predicted interaction effect between State-Trait Anxiety Inventory (STAI) trait score, preceding response type (fast hit, false alarm) in the go/no-go task, and word valence on word categorization response time (RT) in patients with OCD, computed as partial effects from the linear mixed model. RT data were log-transformed for analysis but were back-transformed to ms for data visualization. The plot was generated using the sjPlot package (Version 2.8.11; Lüdecke, 2022). **(B)** Negative correlation between the STAI trait score and the overall priming effect across participants. The overall priming effect refers to the priming effect after false alarms and fast hits and was calculated by subtracting the RT in congruent conditions (positive words after fast hits and negative words after false alarms) from the RT in incongruent conditions (positive words after false alarms and negative words after fast hits) for each participant (Aarts et al., 2012). **(A–B)** Shaded bands represent 95% confidence intervals.

**Table S5**

*Linear Mixed Model Results for Word Categorization Response Time (RT) With OCD-Related Characteristics as Predictors*

|  | Word categorization RT | | | | | | | | | |
| --- | --- | --- | --- | --- | --- | --- | --- | --- | --- | --- |
|  | Patients with OCD | | | | |  | Healthy control participants | | | |
| Fixed effect | *b* | | 95% CI | *t* | *p* | *b* | | 95% CI | *t* | *p* |
| *Obsessive-compulsive symptoms (OCI-R)* | | | | | | | | | | |
| Intercept | 6.46 | | [6.39, 6.53] | 193.95 | **< .001** | 6.41 | | [6.33, 6.50] | 153.07 | **< .001** |
| FA − FH | 0.16 | | [0.10, 0.21] | 6.06 | **< .001** | 0.18 | | [0.11, 0.24] | 5.21 | **< .001** |
| Valence (Pos − Neg) | | 0.00 | [−0.04, 0.04] | 0.01 | .996 | 0.06 | | [0.01, 0.11] | 2.50 | **.018** |
| OCI-R | −0.00 | | [−0.01, 0.01] | −0.49 | .628 | −0.00 | | [−0.02, 0.01] | −0.24 | .814 |
| FA − FH × Valence | 0.14 | | [0.09, 0.19] | 5.62 | **< .001** | 0.22 | | [0.13, 0.30] | 5.19 | **< .001** |
| FA − FH × OCI-R | −0.00 | | [−0.01, 0.00] | −1.30 | .205 | 0.00 | | [−0.01, 0.02] | 0.52 | .607 |
| Valence × OCI-R | −0.00 | | [−0.01, 0.00] | −0.87 | .391 | −0.00 | | [−0.01, 0.01] | −0.32 | .755 |
| FA − FH × Valence  × OCI-R | −0.01 | | [−0.01, −0.00] | −2.44 | **.021** | −0.00 | | [−0.02, 0.01] | −0.08 | .934 |
| *Trait anxiety (STAI trait)* | | | | | | | | | | |
| Intercept | 6.46 | | [6.39, 6.53] | 194.97 | **< .001** | 6.41 | | [6.33, 6.49] | 163.54 | **< .001** |
| FA − FH | 0.16 | | [0.10, 0.21] | 6.00 | **< .001** | 0.18 | | [0.11, 0.24] | 5.27 | **< .001** |
| Valence | 0.00 | | [−0.04, 0.04] | 0.05 | .958 | 0.06 | | [0.01, 0.11] | 2.51 | **.018** |
| STAI | 0.00 | | [−0.00, 0.01] | 0.68 | .505 | 0.01 | | [−0.00, 0.02] | 1.93 | .064 |
| FA − FH × Valence | 0.14 | | [0.09, 0.19] | 5.53 | **< .001** | 0.22 | | [0.13, 0.30] | 5.20 | **< .001** |
| FA − FH × STAI | −0.00 | | [−0.01, 0.00] | −0.89 | .380 | 0.00 | | [−0.01, 0.01] | 0.86 | .399 |
| Valence × STAI | −0.00 | | [−0.00, 0.00] | −0.62 | .539 | 0.00 | | [−0.00, 0.01] | 0.53 | .604 |
| FA − FH × Valence  × STAI | −0.00 | | [−0.01, 0.00] | −2.03 | .053 | 0.00 | | [−0.01, 0.01] | 0.46 | .653 |
| *Trait worry (PSWQ)* | | | | | | | | | | |
| Intercept | 6.46 | | [6.39, 6.53] | 195.05 | **< .001** | 6.41 | | [6.33, 6.50] | 153.40 | **< .001** |
| FA − FH | 0.16 | | [0.11, 0.21] | 6.19 | **< .001** | 0.18 | | [0.11, 0.24] | 5.22 | **< .001** |
| Valence | 0.00 | | [−0.04, 0.04] | 0.04 | .968 | 0.06 | | [0.01, 0.11] | 2.55 | **.016** |
| PSWQ | −0.00 | | [−0.01, 0.00] | −0.76 | .451 | −0.00 | | [−0.01, 0.01] | −0.35 | .731 |
| FA − FH × Valence | 0.14 | | [0.09, 0.19] | 5.25 | **< .001** | 0.22 | | [0.13, 0.30] | 5.21 | **< .001** |
| FA − FH × PSWQ | −0.00 | | [−0.01, 0.00] | −1.51 | .142 | −0.00 | | [−0.01, 0.01] | −0.46 | .650 |
| Valence × PSWQ | 0.00 | | [−0.00, 0.00] | 0.38 | .704 | 0.00 | | [−0.00, 0.01] | 1.08 | .293 |
| FA − FH × Valence  × PSWQ | −0.00 | | [−0.01, 0.00] | −0.52 | .610 | 0.00 | | [−0.01, 0.01] | 0.09 | .930 |

*Note.* Models were calculated separately for each group since otherwise effects of trait measures are confounded by group differences. Random effects were specified as uncorrelated due to non-convergence of the models with the maximal random-effects structure. Estimates of the fixed effects (regression coefficients *b*) are on the log scale. Trait measures are group-mean centered. Boldface *p* values represent statistical significance (*p* < .05). No. of observations: 3,660 (in the patient group) and 3,357 (in the control group). OCD = obsessive-compulsive disorder; CI = confidence interval; OCI-R = Obsessive-Compulsive Inventory-Revised; FA = false alarm; FH = fast hit; Pos = positive; Neg = negative; STAI = State-Trait Anxiety Inventory; PSWQ = Penn State Worry Questionnaire.

**Table S6**

*Correlations Between OCD-Related Characteristics and the Overall Priming Effect After False Alarms and Fast Hits*

|  | Patients with OCD  (*n* = 28) | | |  | | Healthy control participants  (*n* = 28) | | |
| --- | --- | --- | --- | --- | --- | --- | --- | --- |
| Characteristic | *r*(26) | 95% CI | *p* | | *r*(26) | | 95% CI | *p* |
| OCD symptoms (OCI-R) | −.41 | [−.68, −.05] | **.029** | | −.05 | | [−.42, .33] | .790 |
| Trait anxiety (STAI trait) | −.34 | [−.63, .04] | .078 | | .15 | | [−.24, .49] | .459 |
| Trait worry (PSWQ) | −.22 | [−.55, .17] | .264 | | .02 | | [−.35, .39] | .903 |

*Note.* Pearson correlation coefficients are reported. Boldface *p* values represent statistical significance (*p* < .05). OCD = obsessive-compulsive disorder; OCI-R = Obsessive-Compulsive Inventory-Revised; STAI = State-Trait Anxiety Inventory; PSWQ = Penn State Worry Questionnaire.

**Table S7**

*Correlations Between Questionnaire Scores*

|  | Patients with OCD  (*n* = 28) | | |  | | Healthy control participants  (*n* = 28) | | |
| --- | --- | --- | --- | --- | --- | --- | --- | --- |
| Questionnaire scores | *r*(26) | 95% CI | *p* | | *r*(26) | | 95% CI | *p* |
| OCI-R & STAI trait | 0.53 | [0.19, 0.75] | **.004** | | 0.39 | | [0.01 0.66] | **.043** |
| OCI-R & PSWQ | 0.33 | [−0.05, 0.62] | .091 | | 0.40 | | [0.03 0.67] | **.034** |
| STAI trait & PSWQ | 0.62 | [0.32, 0.81] | **< .001** | | 0.42 | | [0.06, 0.69] | **.025** |

*Note.* Pearson correlation coefficients are reported. Boldface *p* values represent statistical significance (*p* < .05). OCD = obsessive-compulsive disorder; OCI-R = Obsessive-Compulsive Inventory-Revised; STAI = State-Trait Anxiety Inventory; PSWQ = Penn State Worry Questionnaire.

**Table S8**

*Random Effects (SDs) of the Linear Mixed Models on Error-Related Negativity (ERN) and Correct-Response Negativity (CRN) in the Go/No-Go Task*

| Random effect | ERN | CRN |
| --- | --- | --- |
| Participants (*N* = 56) |  |  |
| Intercept | 4.55 | 3.41 |
| Residual | 8.50 | 7.98 |

*Note.* Models with the maximal random-effects structure were used.

**Table S9**

*Random Effects (SDs) of the Linear Mixed Model on Word Categorization Response Time (RT) With Error-Related Negativity (ERN) and Correct-Response Negativity (CRN) as Predictor*

|  | Word categorization RT | |
| --- | --- | --- |
| Random effect | With ERN as predictor | With CRN as predictor |
| Participants (*N* = 56) |  |  |
| Intercept | 0.24 | 0.17 |
| Valence (Pos − Neg) | 0.17 | 0.08 |
| ERP ^a^ | – | – |
| Valence × ERP ^a^ | – | – |
| Word stimuli (*N* = 60) |  |  |
| Intercept | 0.02 | 0.03 |
| Group (OCD − HC) | 0.02 | – |
| ERP ^a^ | 0.01 | 0.01 |
| Group × ERP ^a^ | – | – |
| Residual | 0.23 | 0.17 |

*Note.* Random effects were specified as uncorrelated due to non-convergence of the models with the maximal random-effects structure. Random effects explaining zero variance were removed and are indicated by dashes. Pos = positive; Neg = negative; ERP = event-related potential; OCD = obsessive-compulsive disorder; HC = healthy control.

^a^ ERP refers to ERN or CRN as predictor in the respective model.

**Table S10**
*Linear Mixed Model Results for Word Categorization Response Time (RT) and Generalized Linear Mixed Model Results for Word Categorization Accuracy With Session Number as Covariate*

|  | Word categorization RT | | | |  | | Word categorization accuracy | | | |
| --- | --- | --- | --- | --- | --- | --- | --- | --- | --- | --- |
| Fixed effect | *b* | 95% CI | *t* | *p* | | OR | | 95% CI | *z* | *p* |
| Intercept | 6.44 | [6.39, 6.49] | 272.93 | **< .001** | | 21.31 | | [16.79, 27.05] | 25.14 | **< .001** |
| FH − SH | −0.02 | [−0.03, −0.01] | −3.09 | **.003** | | 0.86 | | [0.75, 1.00] | −2.01 | **.045** |
| FA − FH | 0.17 | [0.13, 0.21] | 8.31 | **< .001** | | 0.70 | | [0.54, 0.91] | −2.66 | **.008** |
| IR − FA | −0.02 | [−0.06, 0.03] | −0.71 | .482 | | 2.83 | | [2.19, 3.65] | 7.95 | **< .001** |
| Valence (Pos − Neg) | 0.01 | [−0.02, 0.04] | 0.77 | .445 | | 0.55 | | [0.38, 0.79] | −3.18 | **.001** |
| Group (OCD − HC) | 0.03 | [−0.06, 0.13] | 0.74 | .466 | | 1.27 | | [0.85, 1.91] | 1.15 | .248 |
| Session (2 − 1) | −0.07 | [−0.16, 0.02] | −1.53 | .132 | | 0.88 | | [0.59, 1.31] | −0.61 | .539 |
| FH − SH × Valence | −0.02 | [−0.04, −0.00] | −2.29 | **.027** | | 1.07 | | [0.80, 1.42] | 0.47 | .639 |
| FA − FH × Valence | 0.18 | [0.14, 0.22] | 8.31 | **< .001** | | 0.09 | | [0.05, 0.17] | −8.12 | **< .001** |
| IR − FA × Valence | −0.10 | [−0.14, −0.06] | −5.41 | **< .001** | | 5.56 | | [3.29, 9.38] | 6.42 | **< .001** |
| FH − SH × Group | 0.01 | [−0.01, 0.03] | 1.04 | .305 | | 0.83 | | [0.63, 1.08] | −1.38 | .167 |
| FA − FH × Group | −0.02 | [−0.10, 0.06] | −0.42 | .675 | | 0.90 | | [0.54, 1.49] | −0.41 | .683 |
| IR − FA × Group | −0.02 | [−0.12, 0.07] | −0.54 | .591 | | 1.17 | | [0.71, 1.94] | 0.62 | .537 |
| Valence × Group | −0.04 | [−0.09, −0.00] | −2.10 | **.041** | | 1.87 | | [1.06, 3.30] | 2.15 | **.031** |
| FH − SH × Session | −0.01 | [−0.03, 0.02] | −0.45 | .652 | | 0.96 | | [0.73, 1.25] | −0.32 | .752 |
| FA − FH × Session | −0.02 | [−0.10, 0.06] | −0.60 | .551 | | 1.97 | | [1.19, 3.25] | 2.65 | **.008** |
| IR − FA × Session | 0.02 | [−0.07, 0.11] | 0.45 | .654 | | 0.49 | | [0.30, 0.81] | −2.79 | **.005** |
| Valence × Session | 0.03 | [−0.01, 0.08] | 1.60 | .116 | | 0.49 | | [0.29, 0.84] | −2.58 | **.010** |
| Group × Session | −0.01 | [−0.20, 0.17] | −0.16 | .875 | | 0.80 | | [0.36, 1.77] | −0.56 | .578 |
| FH − SH × Valence × Group | −0.00 | [−0.04, 0.03] | −0.16 | .872 | | 1.03 | | [0.61, 1.76] | 0.12 | .907 |
| FA − FH × Valence × Group | −0.08 | [−0.17, 0.00] | −1.94 | .058 | | 2.29 | | [0.77, 6.80] | 1.49 | .136 |
| IR − FA × Valence × Group | 0.06 | [−0.01, 0.14] | 1.68 | .100 | | 0.68 | | [0.24, 1.93] | −0.73 | .466 |
| FH − SH × Valence × Session | −0.04 | [−0.08, −0.01] | −2.53 | **.015** | | 1.07 | | [0.63, 1.81] | 0.24 | .812 |
| FA − FH × Valence × Session | −0.03 | [−0.12, 0.05] | −0.77 | .443 | | 0.48 | | [0.16, 1.41] | −1.34 | .181 |
| IR − FA × Valence × Session | 0.06 | [−0.02, 0.13] | 1.58 | .120 | | 2.58 | | [0.91, 7.34] | 1.78 | .075 |
| FH − SH × Group × Session | −0.02 | [−0.06, 0.03] | −0.87 | .389 | | 1.44 | | [0.84, 2.45] | 1.34 | .181 |
| FA − FH × Group × Session | 0.01 | [−0.15, 0.18] | 0.19 | .854 | | 0.48 | | [0.18, 1.30] | −1.44 | .150 |
| IR − FA × Group × Session | 0.05 | [−0.13, 0.24] | 0.60 | .552 | | 2.42 | | [0.90, 6.52] | 1.74 | .081 |
| Valence × Group × Session | −0.03 | [−0.11, 0.06] | −0.60 | .550 | | 0.69 | | [0.23, 2.03] | −0.68 | .498 |
| FH − SH × Valence × Group × Session | −0.02 | [−0.08, 0.05] | −0.46 | .647 | | 0.58 | | [0.20, 1.66] | −1.02 | .308 |
| FA − FH × Valence × Group × Session | 0.15 | [−0.03, 0.32] | 1.71 | .093 | | 0.17 | | [0.02, 1.43] | −1.63 | .103 |
| IR − FA × Valence × Group × Session | −0.10 | [−0.25, 0.05] | −1.32 | .192 | | 2.27 | | [0.29, 17.67] | 0.78 | .435 |

*Note.* Random effects were specified as uncorrelated due to non-convergence of the models with the maximal random-effects structure. Estimates of the model on RT (regression coefficients *b*) are on the log scale. Estimates of the model on accuracy reflect the probability of a correct response as odds ratio, and *p* values were calculated using Wald *Z* tests. Boldface *p* values represent statistical significance (*p* < .05). No. of observations: 24,189 (RT) and 26,123 (accuracy). CI = confidence interval; FH = fast hit; SH = slow hit; FA = false alarm; IR = inhibited response; Pos = positive; Neg = negative; OCD = obsessive-compulsive disorder; HC = healthy control.

**Table S11**

*Linear Mixed Model Results for Word Categorization Response Time (RT) With Error-Related Negativity (ERN) and Correct-Response Negativity (CRN) as Predictor and Session Number as Covariate*

|  | Word categorization RT | | | | | | | | | |
| --- | --- | --- | --- | --- | --- | --- | --- | --- | --- | --- |
|  | With ERN as predictor | | | |  | | With CRN as predictor | | | |
| Fixed effect | *b* | 95% CI | *t* | *p* | | *b* | | 95% CI | *t* | *p* |
| Intercept | 6.52 | [6.46, 6.59] | 197.06 | **< .001** | | 6.35 | | [6.31, 6.40] | 269.82 | **< .001** |
| Valence (Pos − Neg) | 0.12 | [0.07, 0.18] | 4.44 | **< .001** | | −0.06 | | [−0.08, −0.03] | −4.27 | **< .001** |
| Group (OCD − HC) | 0.04 | [−0.09, 0.17] | 0.59 | .555 | | 0.05 | | [−0.04, 0.15] | 1.16 | .253 |
| ERP ^a^ | 0.00 | [−0.01, 0.01] | 0.43 | .665 | | −0.01 | | [−0.01, −0.00] | −2.25 | **.027** |
| Session (2 − 1) | −0.09 | [−0.22, 0.04] | −1.33 | .189 | | −0.06 | | [−0.16, 0.03] | −1.35 | .182 |
| Valence × Group | −0.09 | [−0.20, 0.01] | −1.76 | .084 | | −0.03 | | [−0.07, 0.02] | −1.10 | .274 |
| Valence × ERP ^a^ | 0.00 | [−0.02, 0.02] | 0.21 | .833 | | −0.01 | | [−0.02, 0.01] | −0.93 | .357 |
| Group × ERP ^a^ | 0.01 | [−0.01, 0.03] | 1.20 | .229 | | −0.01 | | [−0.02, 0.00] | −1.39 | .166 |
| Valence × Session | −0.01 | [−0.12, 0.10] | −0.19 | .851 | | 0.02 | | [−0.02, 0.07] | 1.06 | .295 |
| Group × Session | −0.02 | [−0.29, 0.24] | −0.16 | .872 | | −0.05 | | [−0.23, 0.14] | −0.49 | .627 |
| ERP ^a^ × Session | 0.00 | [−0.02, 0.02] | 0.30 | .764 | | −0.01 | | [−0.02, 0.00] | −1.33 | .185 |
| Valence × Group × ERP ^a^ | 0.01 | [−0.03, 0.05] | 0.52 | .605 | | 0.01 | | [−0.01, 0.03] | 0.61 | .541 |
| Valence × Group × Session | 0.06 | [−0.15, 0.28] | 0.60 | .554 | | −0.07 | | [−0.16, 0.03] | −1.44 | .156 |
| Valence × ERP ^a^ × Session | 0.00 | [−0.03, 0.04] | 0.20 | .840 | | −0.00 | | [−0.02, 0.02] | −0.36 | .716 |
| Group × ERP ^a^ × Session | −0.01 | [−0.04, 0.03] | −0.29 | .770 | | 0.02 | | [−0.01, 0.04] | 1.43 | .153 |
| Valence × Group × ERP ^a^ × Session | 0.01 | [−0.07, 0.08] | 0.18 | .856 | | −0.04 | | [−0.08, 0.01] | −1.71 | .088 |

*Note.* ERN amplitude and CRN amplitude were entered as *z*-standardized continuous predictors in the models including only false alarm trials and fast hit trials, respectively. Random effects were specified as uncorrelated due to non-convergence of the models with the maximal random-effects structure. Estimates of the fixed effects (regression coefficients *b*) are on the log scale. Boldface *p* values represent statistical significance (*p* < .05). No. of observations: 1,992 (model with ERN) and 5,025 (model with CRN). CI = confidence interval; Pos = positive; Neg = negative; OCD = obsessive-compulsive disorder; HC = healthy control; ERP = event-related potential.

^a^ ERP refers to ERN or CRN as predictor in the respective model.

**References**

Aarts, K., De Houwer, J., & Pourtois, G. (2012). Evidence for the automatic evaluation of self-generated actions. *Cognition*, *124*(2), 117–127. <https://doi.org/10.1016/j.cognition.2012.05.009>

Aarts, K., De Houwer, J., & Pourtois, G. (2013). Erroneous and correct actions have a different affective valence: Evidence from ERPs. *Emotion*, *13*(5), 960–973. <https://doi.org/10.1037/a0032808>

Balzus, L., Klawohn, J., Elsner, B., Schmidt, S., Brandt, S. A., & Kathmann, N. (2022). Non-invasive brain stimulation modulates neural correlates of performance monitoring in patients with obsessive-compulsive disorder. *NeuroImage: Clinical*, *35*, Article 103113. <https://doi.org/10.1016/j.nicl.2022.103113>

Balzus, L., Klawohn, J., & Kathmann, N. (2021). Feeling bad about being wrong: Affective evaluation of performed actions and its trial-by-trial relation to autonomic arousal. *Emotion*, *21*(7), 1402–1416. <https://doi.org/10.1037/emo0000995>

Green, P., & MacLeod, C. J. (2016). SIMR: An R package for power analysis of generalized linear mixed models by simulation. *Methods in Ecology and Evolution*, *7*(4), 493–498. <https://doi.org/10.1111/2041-210X.12504>

Lüdecke, D. (2022). *sjPlot: Data visualization for statistics in social science* (Version 2.8.11) [Computer software]. <https://CRAN.R-project.org/package=sjPlot>

Morey, R. D. (2008). Confidence intervals from normalized data: A correction to Cousineau (2005). *Tutorials in Quantitative Methods for Psychology*, *4*(2), 61–64. <https://doi.org/10.20982/tqmp.04.2.p061>
